# The Impact of Video-Based Educational Interventions on Cervical Cancer, Pap Smear and HPV Vaccines

**DOI:** 10.3389/fpubh.2021.681319

**Published:** 2021-07-07

**Authors:** Emmanuel Kwateng Drokow, Clement Yaw Effah, Clement Agboyibor, Evans Sasu, Cecilia Amponsem-Boateng, Gloria Selorm Akpabla, Hafiz Abdul Waqas Ahmed, Kai Sun

**Affiliations:** ^1^Department of Radiation Oncology, Zhengzhou University People's Hospital and Henan Provincial People's Hospital Henan, Zhengzhou, China; ^2^College of Public Health, Zhengzhou University, Zhengzhou, China; ^3^School of Pharmaceutical Sciences, Zhengzhou University, Zhengzhou, China; ^4^Department of Radiotherapy, National Centre for Radiotherapy and Nuclear Medicine, Korle Bu Teaching Hospital, Accra, Ghana; ^5^Department of Epidemiology and Biostatistics, College of Public Health, Zhengzhou University, Zhengzhou, China; ^6^Department of Internal Medicine, Tianjin Medical University, Tianjin, China; ^7^Department of Haematology, Zhengzhou University People's Hospital and Henan Provincial People's Hospital Henan, Zhengzhou, China

**Keywords:** cervical cancer, video based, educational intervention, pap smear test, human papillomavirus

## Abstract

**Background:** Video-based interventions have the potential to contribute to long-lasting improvements in health-seeking behaviours. Ghana's upsurge rate of information and communication technology usage presents an opportunity to improve the awareness of HPV vaccination and screening rates of cervical cancer among women in Ghana. This research aimed to assess the impact of video-based educational intervention centred on the Health Belief and Transtheoretical Models of behavioural changes in promoting HPV vaccination, cervical carcinoma awareness and willingness to have Pap smear test (PST) among women in Ghana.

**Methods:** To achieve the intended sample size, convenient, purposive and stratified random sampling techniques were used. SPSS v. 23.0 was used in the data analysis. Percentages and frequencies were used to represent participants' demographic characteristics, knowledge of (1) cervical carcinoma, (2) human papillomavirus vaccine, and (3) Pap smear test. The chi-square test by McNemar was employed to evaluate variations in the post- and pre-intervention responses. A *p*-value < 0.05 was considered statistically significant. The level of significance was adjusted owing to multiple comparisons by using the Bonferroni's correction.

**Results:** Before the intervention, 84.2% of the participant had some knowledge or information about cervical cancer, but after the intervention, 100% of the participant became aware of cervical cancer which represents 15.8% increment at a *P* < .001. The willingness to have a pap smear test increased from 35.8% to 94.2% (df = 58.4%, *P* < .001) after the educational intervention. The willingness to be vaccinated increased from 47.5% to 81.7% (df = 34.2%, *P* < .001) after the educational intervention. Six months after the intervention, participants were followed-up. 253 (42.2%) participants had gone for cervical cancer screening (Pap smear test) while 347 (57.8%) participants had not been screened. In terms of HPV vaccination, 192 participants (32.0%) had begun their HPV vaccination cycle.

**Conclusion:** The study results show that health education, using videos, may be influential in perception changing, self-efficacy improvement and the understanding of cervical carcinoma screening and HPV vaccination.

## Introduction

The prevalence of cervical cancer (CC) tends to draw the attention of researchers and primary healthcare providers. The Globocan (2018) report rated cervical cancer as the fourth most commonly diagnosed cancer among women with annual new registered cases of 569,847 and 311,365 deaths worldwide (1). Cervical cancer ranks second after breast cancer in Africa with a high mortality and incidence rate of 81,687 and 119,284, respectively, and the age-standardisation rate is 20.0 and 27.6 per 100,000 women. Ghana is a low-middle-income country with annual diagnosed cervical cancer cases of 3,151 and 2,119 deaths (2).

The epidemiology of cervical cancer has been associated with infections with human papillomavirus (HPV). The International Agency for Research on Cancer monographs has categorised 12 distinct types of oncogenes as group 1 carcinogens with HPV 18 and 16 been the predominant once (3, 4). Other risk factors mentioned in literature include long term usage of oral contraceptives, early marriage, multiparity, immunocompromised, insufficient vegetable and fruit intake, overweight/obesity and smoking (5). Presently, there is no nation-wide cervical cancer screening programme in Ghana. Nevertheless, the screening age in Ghana is from 25–64 years at a screening interval of 3–5 years (6). Women between the ages of 25 to 45 are mostly screened using visual inspection with acetic acid (VIA) whiles cytology (Pap smear test) is used in screening women who are above 45 years (6). Educational barriers, lack of awareness and knowledge toward HPV vaccine and pap smear test, screening and vaccination cost are some possible obstacles toward the acceptability of HPV vaccination and Pap smear test. For instance, the price of Cervarix a bivalent HPV vaccine used in Ghana cost GHC320 ($60) per jab, which is prohibitive for many females especially those in rural areas. It is obvious that education strategies and effective screening are needed in Ghana.

The feasibility, sustainability, effectiveness and implementation of preventive strategies toward cervical cancer and HPV infections in developed and industrialised nations has emerged useful (7). Considering the well-structured initiatives on HPV vaccination and screening of cervical cancer in many nations, a substantial decrease in mortality and prevalence of cervix carcinoma has been accomplished to some degree (8). One such intervention is the primary vaccination to prevent human papillomavirus infections. The human papillomavirus bivalent vaccine Cervarix^®^ as a prophylactic vaccine has demonstrated to protect younger women who are yet to be infected with HPV 18 and 16 (9). The Cervarix vaccine is known to be highly effective, immunogenic and safe in preventing about 70% of cervical carcinoma worldwide, (9–18) usually before being infected with HPV and also before becoming sexually active particularly among younger women aged 9–26 (15).

The Expanded Program of Immunization (EPI) established by the World Health Organization has effectively and efficiently increased the availability and accessibility of childhood vaccines. It has attained more comprehensive coverage globally, including African nations. Nevertheless, the lack of knowledge and the low level of awareness on HPV vaccination may have a negative influence on HPV vaccination programs in Africa (19, 20). Hence, it is imperative to assess effective initiatives to improve uptake of the HPV vaccine.

The prevention of ailments with significant mortality and morbidity can be achieved through modifying and developing health behaviour (21). Educational interventions that promote healthier attitudes may help to promote the welfare of people by promoting healthy lifestyles. Health knowledge can be impacted through various educational programs, such as web-based applications, oral counselling via face-to-face, videos and printed materials (22–24). The day-to-day utilisation of the internet in the health sector has upsurge in recent years, and individuals can readily obtain any information. Web-based education (WBE) with incredible visual and audio educational resources, gives individuals the opportunity to obtain information anywhere there is an internet accessibility, and allows individuals to revisit the website, to study and understand (25). Web-based education is indicated to be cost-effective, satisfactory, suitable and effective. WBE helps in reaching a large population, improves healthy behaviours and increases the depth of knowledge (26, 27).

A video-based educational intervention on smoking prevention and human immunodeficiency virus projects in the United States of America resulted in significant costs reduction (28, 29). Furthermore, VBEI offers standardisation of education among the educators' boards when it comes to information presentation (30). Third, VBEI is more receptive to people with lower levels of health literacy (31). Again, VBEI can be administered in different formats, including downloadable media files, streaming videos, videotape, and versatile disc/ digital video. Video-sharing educational videos can reach a wider crowd via social media (32–34). Considering the facts that social media channels like YouTube function as a valuable medium for health information delivery, there seems to be little evidence about its impact on improving knowledge and awareness of pap smear, human papillomavirus vaccination and its role in decreasing the challenges in cervical cancer screening. Ampofo et al. concluded that video-based educational intervention is an effective approach for improving cervical cancer awareness (35). Karakuş et al. also reported that web-based intervention was observed to increase the Pap smear test behaviour among Indonesian teachers (25).

Furthermore, Ebu et al. suggested that leaflets, videos and lectures as a form of educational intervention can sharpen an individual's knowledge and perceptions on cervix carcinoma and it's screening (36). A research carried out in Cameroon and Kenya demonstrated a high level of acceptability and awareness of the human papillomavirus vaccine, and this was attributable to a community-based educational program (36–38). Ghana's upsurge rate of communication and information technologies usage presents an opportunity to improve awareness of HPV vaccination and screening rates of cervical cancer among women in Ghana. It may also be utilised to sensitise women in Ghana to screen for cervical cancer, thereby improving their screening attitudes and also to increase the awareness of primary preventive measures.

However, to the authors' best of knowledge, no research has exclusively investigated the impact of video-based educational interventions on the awareness and knowledge level on Cervical Cancer, Pap smear and HPV Vaccines. This research aimed to assess the impact of a video-based educational intervention centred on the Health Belief and Transtheoretical Models of behavioural changes in promoting HPV vaccination, cervical carcinoma awareness and willingness to have Pap smear test (PST) among women in Ghana. We hypothesised that an educational intervention using videos would improve participants' knowledge and understanding of Pap smear test, cervix carcinoma and HPV vaccine. We also hypothesised that VBEI can decrease the barriers to cervical cancer screening and HPV vaccination.

## Methods

### Study Population

This population-based cross-sectional survey on the impact of video-based educational interventions on cervical cancer, pap smear and HPV vaccines was carried between the years 2019 to 2020. The sampling methods used in this survey were stratified random sampling, purposive and convenient sampling. Participants were invited to partake in the survey via a formal electronic and paper invitation. The email addresses of those who were reached electronically were collected via a previous exercise conducted within the Municipalities. We used convenient sampling for participants we had their emails. In addition, participants who did not have internet accessibility or for whom we did not have their email addresses were recruited by means of smaller local groups within the communities, home visits and church groups. The population (i.e., the churches, homes and communities) were categorised into three strata (3) and random sampling was employed so that every individual in the communities, homes and church groups had an equitable opportunity of participating in the survey. The purposive sampling method was used to ensure that all respondents in the survey met the inclusion criteria. The target population were residing at Takoradi, Sunyani, Kumasi and Accra. The selection of the four cities were centered on their population density and also with the objective of achieving a representative sample of the country's population.

### Inclusion and Exclusion Criteria

The inclusion criteria were; (i) any sound-minded female Ghanaian resident (ii) must be 18 years and above, (iii) not deaf and dumb, (iv) women with no history of HPV vaccination and Pap smear test, (v) women who owned and used any ICT device. All participants who did not meet these criteria were excluded from this study. The criteria for exclusion involved females with history of Pap smear test, females diagnosed with cervix carcinoma and individuals who did not give their consent. Responses to the above exclusion query were acquired by asking the respondents in an interview prior to the questionnaire being administered.

### Study Design

The questionnaires employed in this study is a modified version of the one used in our previous study after it was approved by experts (39). An advisory committee of two experts in research methodology, gynaecology and obstetrics, and oncology evaluated the questionnaire legitimacy and soundness prior to the pilot test. According to the expert's comments, three questions relating to signs and symptoms were revised, and two questions not relevant to the subject were omitted. A pilot study was then performed with 30 participants on the pre-final prototype to assess the clarity of the questionnaire. Results from the pilot and current study showed that the Cronbach alpha value was **0.916**. The Cronbach's alpha assesses a given dataset's consistency or internal reliability. The questionnaire-based study was conducted after all respondents provided written consent, with their confidentiality and anonymity retained. The sample size was calculated by using the formula of the minimum sample size; thus, “*n* = Z^2^P(1-P)/d2; where, *n* = sample size, Z = z statistic for a level of confidence. For the level of confidence of 95%, which is conventional, the Z value is 1.96. *P* = expected prevalence or proportion (in proportion of one; if 46%, *P* = 0.46), and *d* = precision (in proportion of one, if 5%, *d* = 0.05).” The estimated sample size was 382 using an expected prevalence or proportion(p) of 46%; *P* = 0.46, considering a 95% confidence interval (CI) and a 5% marginal error. To account for heterogeneity in the target group and also ensure that maximum responses were obtained, we increased the sampling size and targeted about 645 participants.

On the day of administering the questionnaire, all the selected respondents answered a baseline questionnaire. No control group was involved in this study because it was a pre-post-study. Video-based educational interventions were used to educate the participants.

### Educational Intervention

The Health Belief Model (HBM) and the Transtheoretical Model (TTM) were the two principal models in health behaviour change theories on which the interventional study was centred. These models have been utilised effectively in a similar setting to promote positive cancer screening attitude (40). The Transtheoretical Model evaluates the readiness of an individual to make positive behavioural changes and offers an elaborated and systematic plan of action to aid the individual progress across the Stages of Changes (pre-contemplation, contemplation, preparation, action, maintenance, and termination) in the TTM under the hypothesis that an individual will follow a healthy protective behaviour attributable to increase in knowledge and high awareness level (41). The Health Belief Model was used in the intervention to mitigate barriers and demonstrate the advantages of screening since the HBM aids in identifying barriers and to promote positive behavioural changes (42). Several videos describing the Pap smear, HPV vaccine and the incidence, risk factors and symptoms of cervix carcinoma were downloaded from YouTube. Considering the aim of the study, three videos that were deemed appropriate were selected from the collection to create the final video for the intervention. The video further portrayed a pictorial illustration of cervical cancer progression in an individual infected with the Human Papillomavirus as well as the available treatment modalities. An approval from the authors was sought before downloading the videos. It took averagely about 15 min for a participant to watch the interventional video. The video was played twice for clarity purpose since some of the respondents may not have grasps the component of the video for the first time. This was done in every 2 months until the end of the 6months' intervention period. The intervention was conducted by research assistants that included a health educator and licenced nurse practitioner. Question and answer session was conducted after the intervention to further address certain crucial questions about the disease.

### Timing for Evaluating the Effectiveness of the VBEI

The respondents were followed-up 6 months after staging the intervention to complete the endline questionnaire to assess their attitude and knowledge on cervix carcinoma, pap smear test and HPV vaccination. Interviews and the questionnaire were used to evaluate the impact of the VBEI on HPV vaccination and cervical cancer screening.

### Structure of the Survey

The questionnaire design and group selection were centred on Triadic Impact theory (TTI) (30) and Social-Ecological Model (SEM) (31). The Theory of Triadic Influence assumes a “3 × 3 system of environmental, intrapersonal, and interpersonal sources of influence intersected by distal, proximal, and ultimate degrees of influence. The Social-Ecological Model (SEM) takes into consideration public policy, interpersonal, community, institutional and intrapersonal as levels for influencing health-related behaviours. Although these concepts vary in structure and variable interaction, they share several theoretical principles, allowing them to be integrated in this survey. Each survey question in the questionnaire was generated by adopting and modifying questions from previously published articles and was translated and fine-tuned to ensure that people understood the instructions in a comfortable and comprehensible context.

To help the participants respond to the questions quickly and easily, the questionnaire questions covered was categorised into knowledge on HPV vaccine, cervical cancer and Pap smear test (PST) and sociodemographic. The section for cervical cancer was subcategorised into (a) cervical cancer knowledge, (b) symptoms of cervical cancer, and (c) cervical cancer risk factors. If a respondent replied that they were knowledgeable of cervical cancer by saying that they had learned or knew about it, their knowledge of the disease was assessed. Awareness of the risk factors by the respondent included [“Can HPV infection cause cervical cancer,” “long term use of oral contraceptives pills,” “smoking,” “unprotected sexual practices,” “multiparity,” “Immunocompromised/HIV-AIDS,” “early age at marriage,” “Body mass index ≥ 25 kg/m^2^,” “Family history of cervical cancer,” “Having sexually transmitted infections,” “Multiple sexual partners (≥3)”] and symptoms (“lower abdominal pain,” “bleeding after sexual intercourse,” “bleeding in between periods,” “vaginal discharge with foul smell,” “weight loss,” “post-menopausal bleeding,” “asymptomatic,” “Genital warts”) of cervical carcinoma was assessed. PST knowledge was assessed with the phrase, “Have you heard about the Pap smear test?,” “What is a Pap Smear test used for?” “Is there a need for Pap smear test after receiving the HPV vaccine” and “Have you ever had a Pap smear or Pap test?.” The knowledge of the HPV vaccine was assessed in a similar way. Some previous studies have reported these questions (43). Other relevant questions such as “Is HPV infection a sexually transmitted infection?,” “Is a persistent infection of high-risk HPV the leading cause of cervical cancer and other HPV cancer types?,” “Can the HPV vaccine prevent cervical cancer and other HPV cancer types?” and “Must the HPV vaccination be received before the first sexual intercourse?” were preliminary employed in assessing respondents' knowledge regarding HPV and its vaccine. Similar questions employed by past studies (44) in assessing the acceptability of the HPV vaccination included; “Are you willing to vaccinate your current or future children?”, “Are you willing to vaccinate yourself?” and “Would you accept paying for the HPV vaccination?” were also utilised in this survey. Some questions contained three possible responses (don't know, no, yes); nevertheless, the “don't know” response was considered to be a wrong response. Participants were required to select from 12 listed items identified as some possible barriers to screening programs.” The willingness to be screened and vaccinated were evaluated by requesting respondents to choose “No” or “Yes” to “Are you willing to have Pap smear test” and “Are you willing to vaccinate yourself?” respectively. The responses from the respondents were finally classified into one of “the Stages of Change.” The pre-contemplation stage was assigned to respondents who answered “No”. Respondents who responded “Yes” were further asked “Are you willing to have the Pap smear test within the next 180 days?.” “Yes” responses were assigned to “the preparation stage” while the “No” responses were assigned to the Contemplation Stage.

### Data Analysis

SPSS v. 23.0 was used in the data analysis. The respondents' sociodemographic characteristics, knowledge of HPV vaccine, cervical carcinoma, and Pap smear test were represented by percentages and frequencies. The chi-square test by McNemar was employed to evaluate variations in post- and pre-intervention responses. A *p*-value <0.05 was considered statistically significant. The level of significance was adjusted owing to multiple comparisons by using the Bonferroni's correction.

## Results

### Sociodemographic Characteristics

A total of 645 participants answered the survey questions; however, 45 answered the questionnaire incompletely. The remaining 600 participants representing 93.0% response rate, were included in our final analysis. Table 1 shows the sociodemographic characteristics of all the participants. The mean age of all participants was 27 years (range: 19–60) with [Standard Deviation (SD) ±5.5]. Majority of the respondents (69.2%) were between the ages 18-29 years while the remaining 25.5, 3.3, and 2% were between the ages 30–39 years, 40–49 years, and above 50 years, respectively. It is worth noticing that 85.0% of the participants were Christians, and 0.8% were Traditionalist. Regarding educational status, the highest proportion of participants (97.5%) have had college training and above. All the participants have had a means of formal education. In connection with marital status, 81.7% were single while 1.7% were either divorced or widows.

**Table 1:** Sociodemographic features of the participants.

**Sociodemographic characteristics**	**Number (*N* = 600)**	**Percentage (%)**
**Age**		
18–29	415	69.2
30–39	153	25.5
40–49	20	3.3
Above 50	12	2.0
**Religion**		
Christian	510	85.0
Muslim	85	14.2
Traditionalist	5	0.8
**Education**		
Junior high school or below	5	0.8
Senior high school	10	1.7
College/graduate and above	585	97.5
**Occupation**		
Student	430	71.7
Working	90	15.0
Retired	75	12.5
Unemployed	5	0.8
**Marital status**		
Single	490	81.7
Divorced/widow	10	1.7
Married	100	16.6

### Effect of Video-Based Educational Intervention on Awareness and Knowledge of Cervical Cancer and Pap Smear Test

**Figure 1:**
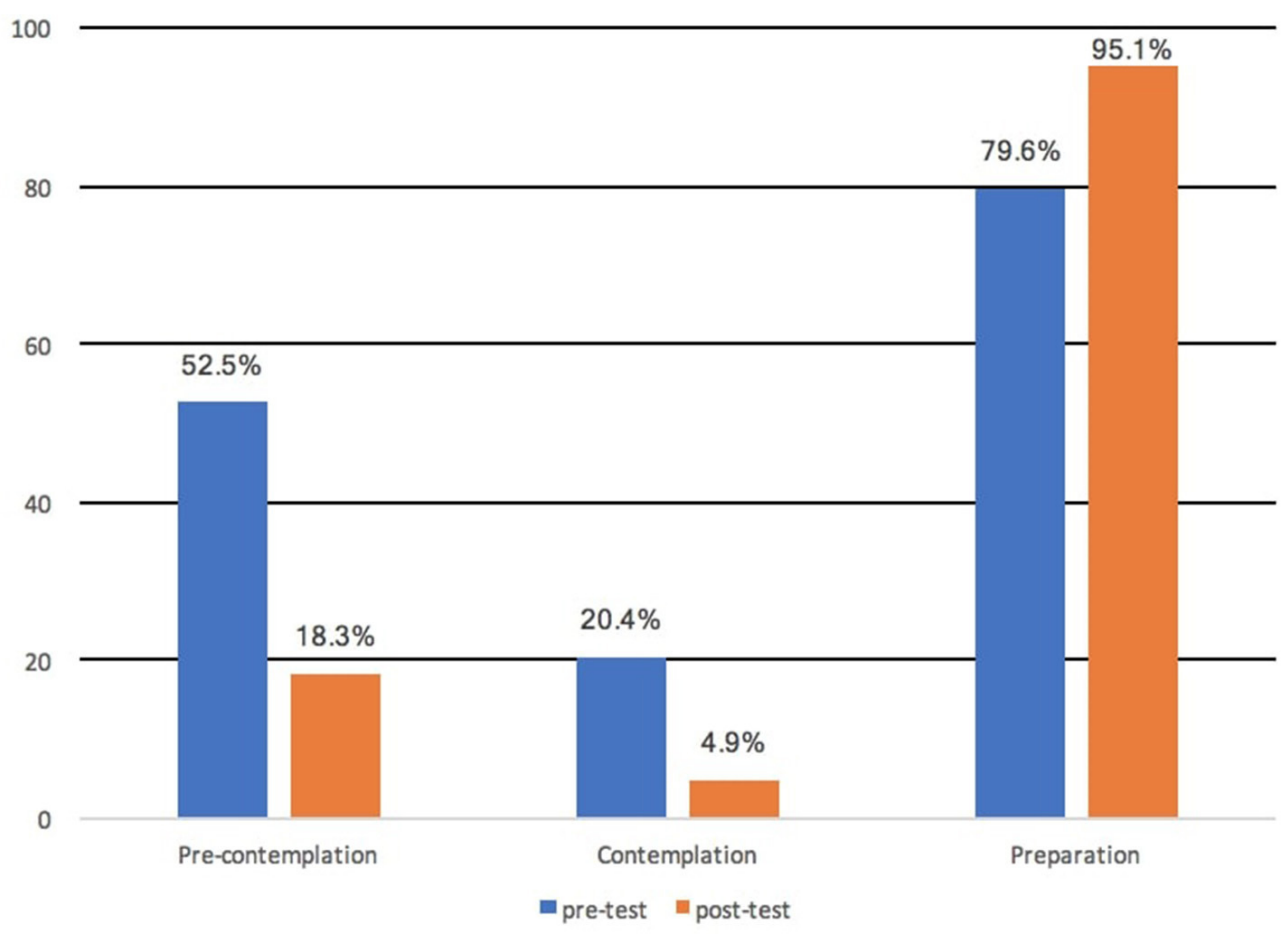
Percentage of participants based on the Stages of Change for the willingness to be screened.

The impact of the interventional study on cervical cancer awareness and knowledge are presented in Table 2. In general, there was a significant difference between the variables used in assessing cervical cancer awareness and knowledge. Before the intervention, 84.2% of the participant had some knowledge or information about cervical cancer, but after the intervention, 100% of the participant became aware of cervical cancer which represents 15.8% increment at a *P* < 0.001. Again, most of the respondents understood that “All women are at risk of developing cervical cancer” with a significant increase in correct responses from 325 (54.2%) to 560 (93.3%) indicating 39.1% rise in correct responses (*P* < 0.001). Regarding Pap smear test, 55.8% responded “Yes” to “Do you know about Pap smear test?,” but after the intervention, 100% responded “Yes” to the same question with the correct responses going up by 44.2% at a significant *P* < 0.001. The willingness to have a pap smear test increased from 35.8 to 94.2% (*df* = 58.4%, *P* < 0.001) after the VBEI. We observed an increase in the intention to have a pap smear test from pre-intervention to post-intervention, with progress via the Stage of Changes from the pre-contemplation stage to the preparation stage (Figure 1). The proportion of respondents in the preparation phase increased from 83.1% at pre-intervention to 96.5% at post-intervention (*P* < 0.001), corresponding to respondents who stated their willingness to have the Pap smear test within the next 6 months.

**Table 2:** Participants' knowledge on cervical cancer and pap smear test after the VEBI.

**Variables**	**Pre-test (*n*, %)**	**Post-test (*n*, %)**	**Difference in %**	***P*-value**
Do you know about cervical cancer? [Yes]	505 (84.2)	600 (100)	15.8	<0.0001
Is cervical cancer one of the most common cancers among females? [Yes]	420 (70.0)	585 (97.5)	27.5	<0.0001
Are all women at risk of developing cervical cancer? [Yes]	325 (54.2)	560 (93.3)	39.1	<0.0001
Is cervical cancer more common in middle age females? [Yes]	370 (61.7)	550 (91.7)	30.0	<0.0001
Is cervical cancer a communicable disease (transmitted by skin contact, sneezing coughing) [No]	525 (87.5)	570 (95.0)	7.5	<0.0001
Do you know about Pap smear test? [Yes]	335 (55.8)	600 (100)	44.2	<0.0001
Pap smear test is used for screening cervical cancer? [Yes]	410 (68.3)	570 (95.0)	26.7	<0.0001
There is no need for Pap smear test after vaccination [No]	145 (24.2)	355 (59.2)	35.0	<0.0001
Are you willing to have a pap smear test? [Yes]	215 (35.8)	565 (94.2)	58.4	<0.0001

### Knowledge of Cervical Cancer Risk Factors and Symptoms After VBEI

Before the educational intervention, 62.5, 57.5, 51.7, 56.7, 42.5, 35.0% knew that “lower abdominal pain, bleeding after sexual intercourse, bleeding in between periods, vaginal discharge with foul smell, post-menopausal bleeding and genital warts,” respectively, were some of the symptoms of cervical carcinoma compared to 95.5, 91.2, 90.8, 95.8, 85.0, and 64.2% after the intervention (Table 3). A significant difference at a *P* < 0.001 was observed across these symptoms when the post and pre-interventional responses were compared. Majority of the respondents understood that Human papillomavirus (HPV) infection is one of the key risk factor in the development of cervix carcinoma with a significant increase in correct responses from 345 (57.5%) to 595 (99.2%) representing 41.7% increase (*P* < 0.001). Other correctly identified risk factors with significant rise in knowledge level regarding such factors include “long term use of oral contraceptives pills”, “unprotected sexual practises,” “Early age at marriage,” “Multiparity,” “Body mass index ≥ 25 kg/m^2^ (Obesity)” and “Family history of cervical cancer” with increment of 45% (from 57.5 to 96.7%), 40.8% (from 53.3 to 94.2%), 40% (from 21.7 to 61.7%), 73.3% (from 20.0 to 93.3%), 55.9% (from 18.3 to 74.2%), and 46.7 (from 50.0 to 96.7%), respectively, with all at a *P* < 0.001 (Table 4).

**Table 3:** Participants' knowledge on cervical cancer risk factors before-and after the VEBI.

**Risk factors**	**Pre-test (*n*, %)**	**Post-test (*n*, %)**	**Difference in %**	***P*-value**
Human papillomavirus (HPV) infection	345 (57.5)	595 (99.2)	41.7	<0.0001
Long term use of oral contraceptives pills	310 (51.7)	580 (96.7)	45.0	<0.0001
Smoking	205 (34.2)	590 (98.3)	64.2	<0.0001
Unprotected sexual practises	320 (53.3)	565 (94.2)	40.8	<0.0001
Multiparity (giving birth to more than 3 children)	120 (20.0)	560 (93.3)	73.3	<0.0001
Immunocompromised/Human immunodeficiency virus/AIDS	235 (39.2)	455 (75.8)	36.6	<0.0001
Early age at marriage	130 (21.7)	370 (61.7)	40.0	<0.0001
Body mass index “25 kg/m2 (Obesity)	110 (18.3)	445 (74.2)	55.9	<0.0001
Poor diet	115 (19.2)	515 (85.8)	66.6	<0.0001
Family history of cervical cancer	300 (50.0)	580 (96.7)	46.7	<0.0001
Having a sexually transmitted infection	325 (54.2)	590 (98.3)	44.2	<0.0001
Multiple sexual partners (≥3)	350 (58.3)	555 (92.5)	34.2	<0.0001

**Table 4:** Participants' knowledge on the symptoms of cervical cancer before-and after the VEBI.

**Symptoms**	**Pre-test (*n*, %)**	**Post-test (*n*, %)**	**Difference in %**	***P*-value**
Lower abdominal pain	375 (62.5)	573 (95.5)	33.0	<0.0001
Bleeding after sexual intercourse	345 (57.5)	547 (91.2)	33.7	<0.0001
Bleeding in between periods	310 (51.7)	545 (90.8)	39.1	<0.0001
Vaginal discharge with foul smell	340 (56.7)	575 (95.8)	39.1	<0.0001
Weight loss	290 (48.3)	495 (82.5)	34.2	<0.0001
Post-menopausal bleeding	255 (42.5)	510 (85.0)	42.5	<0.0001
Asymptomatic (no symptoms)	105 (17.5)	395 (65.8)	48.3	<0.0001
Genital warts	210 (35.0)	385 (64.2)	29.2	<0.0001

### Knowledge on HPV and HPV Vaccine After VBEI

**Figure 2:**
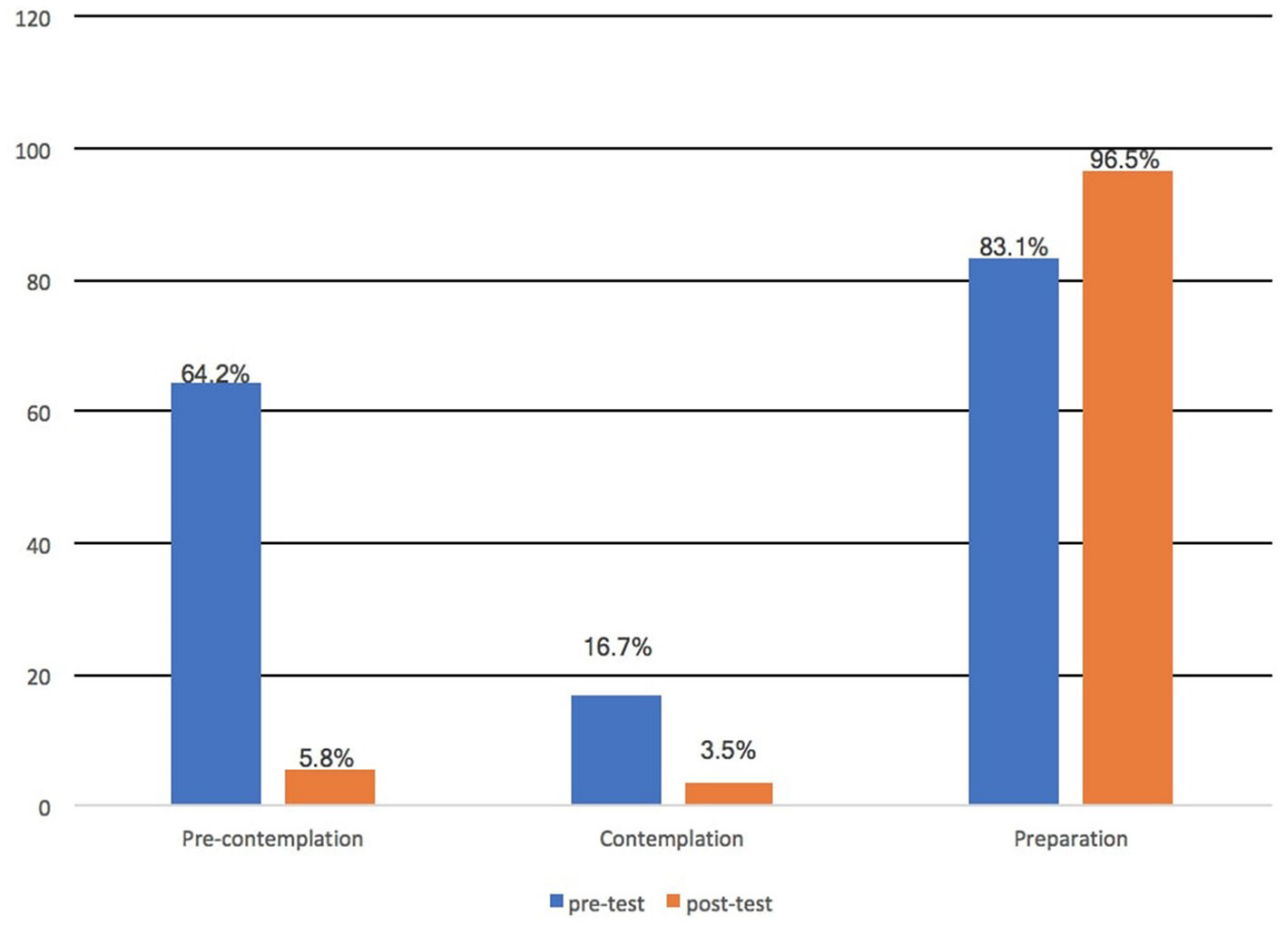
Percentage of participants based on the Stages of Change for the willingness to be vaccinated.

After the intervention, 100% of the participants responded “Yes” to the statement “Have you ever heard of HPV?” compared to the 51.7% who responded Yes to the same question before the intervention, indicating a significant increase of 48.3% in the “Yes” responses (*P* < 0.001). Most participants understood that HPV is sexually transmitted (from 51.7 to 94.2%, *df* = 42.5%, *P* < 0.001) and that HPV infection can go away on its own without treatment (from 10 to 62.5%, *df* = 53.5%, *P* < 0.001). Concerning HPV vaccine, 26.7% responded “Yes” to “Have you heard of the HPV vaccines (Gardasil^®^, Gardasil^®^ 9, and Cervarix^®^)?” in pre-intervention while 100% responded “Yes” to the same question in post-intervention representing 73.3% increase at a significant *P* < 0.001 (Table 5). It is worth noticing that 73.3% was the highest difference between the pre-and post-interventional responses observed in our study. Majority of the respondents became aware of the fact that the HPV vaccine can prevent cervical cancer and other HPV cancer types (from 25.0 to 95.0%, *df* = 70.0%, *P* < 0.001) and also the vaccine can be given to males (from 18.3 to 82.5%, *df* = 64.2%, *P* < 0.001). The willingness to be vaccinated increased from 47.5 to 81.7% (*df* = 34.2%, *P* < 0.001) after the VBEI. We observed an increase in the willingness to be vaccinated from pre-intervention to post-intervention, with progress via the Stage of Changes from the pre-contemplation stage to the preparation stage (Figure 2). The proportion of respondents in the preparation phase increased from 79.6% at pre-intervention to 95.1% at post-intervention (*P* < 0.001), corresponding to respondents who stated their willingness to be vaccinated within the next 6 months. The possible barriers to cervical cancer screening identified by the respondents were “lack of knowledge (70.7%), Poor awareness (59.4%), Lack of understanding about screening procedure (53.4%), Stigma (51.9%), Superstition (37.6%) and family support (30.8%).” Respondent views on the potential use of the videos and their satisfaction with the videos were also evaluated. Our findings showed a high level of video satisfaction among the respondents. Most respondents confirmed that the length of the video is “Right” (89%), and few did not understand some aspect of the video (4.2%).

**Table 5:** Participants' knowledge on HPV and HPV vaccine before-and after the VEBI.

	**Pre-test (*n*, %)**	**Post-test (*n*, %)**	**Difference in %**	***P*-value**
Have you ever heard of HPV? HPV stands for Human Papillomavirus. It is not HIV, HSV, or herpes. [Yes]	310 (51.7)	600 (100)	48.3	<0.0001
Do you think you can get HPV through sexual contact? [Yes]	310 (51.7)	565 (94.2)	42.5	<0.0001
Do you think high-risk HPV can cause cervical cancer? [Yes]	330 (55.0)	590 (98.3)	43.3	<0.0001
Do you think HPV can go away on its own, without any treatment? [Yes]	60 (10.0)	375 (62.5)	52.5	
Have you heard of the HPV vaccine (Gardasil^®^, Gardasil^®^9, and Cervarix^®^)? [Yes]	160 (26.7)	600 (100)	73.3	<0.0001
Can the HPV vaccine prevent cervical cancer and other HPV cancer types? [Yes]	150 (25.0)	570 (95.0)	70.0	<0.0001
Must the HPV vaccination be received before the first sexual intercourse? [Yes]	140 (23.3)	465 (77.5)	54.2	<0.0001
Can the HPV vaccines be given to males? [Yes]	110 (18.3)	495 (82.5)	64.2	<0.0001
Are you willing to vaccinate your current or future children? [Yes]	305 (50.8)	510 (85.0)	34.2	<0.0001
Are you willing to vaccinate yourself? [Yes]	285 (47.5)	490 (81.7)	34.2	<0.0001

Six months after the intervention, participants were followed-up. 253 (42.2%) participants had gone for cervical cancer screening (Pap smear test) while 347 (57.8%) participants had not been screened. In terms of vaccination, 192 participants (32.0%) had begun their HPV vaccination cycle.

## Discussion

The research was geared toward evaluating the impact of VBEI on improving the awareness and knowledge on cervix carcinoma, Pap smear test and HPV vaccines. We hypothesised that an educational intervention using videos would improve participants' knowledge and understanding of Pap smear test, cervix carcinoma and HPV vaccine. Interestingly, in other to achieve our objectives the effect and impact of the educational intervention were totally validated among the respondents in the study. These findings were consistent with the previous study performed by Gottvall et al. (45) where a school-based interventional study was able to improve HPV awareness through classroom teaching and website. Similarly, another study from Kim et al. showed that cervical cancer prevention education was able to educate participants significantly regarding the essence of cervical cancer prevention (16). Our study results indicate an improvement in knowledge concerning cervical carcinoma, Pap smear test and HPV vaccine. One potential explanation of this result may be that respondents acquired certain knowledge and understanding after the interventional study. This confirms the results of a health educational intervention performed in Egypt, Jamaica and Nigeria (46–48). Our results demonstrate that educational intervention centred on theory can be used effectively to increase the willingness of women to be screened for cervical cancer and further improve the knowledge level for cervical carcinoma. Between the pre-test and post-test, there were statistically significant changes in the proportion of correct answers, and most of the respondents progressed from the pre-contemplation stage to the preparation stage. Our findings are consistent with other interventional study using the same methods that have led to a dramatic improvement of cancer awareness and significant improvement in health behaviour (40, 47). The intervention was successful in dispelling many myths regarding symptoms of cervix carcinoma and its causes.

Considering the substantial increase in the level of knowledge about Human papillomavirus as a causative organism of cervix carcinoma, the acceptability of Human papillomavirus vaccination among these women may be easier. Knowing HPV as a high-risk factor of cervix carcinoma may increase HPV vaccination uptake. The HPV vaccines have shown to be safe and very effective in preventing cervical cancer and other HPV cancer types and infections (11, 17, 49–51). This has led to a decrease in the occurrence of abnormal histology and cytology of the cervix (52). Therefore, it is essential to ascertain the understanding of HPV and cervical cancer. It is troubling to note that, despite the proof of the efficacy of HPV vaccination, Ghana has not implemented any nationwide vaccination program.

A notable outcome from our analysis was the fact that the video-based educational intervention massively and significantly improved participant's knowledge of cervix carcinoma risk factors and symptoms. This outcome runs contrary to the findings of Ampofo et al. where educational intervention could not improve the respondent's knowledge of cervix carcinoma risk factors and symptoms (35). The difference can be attributed to the fact that the interventional videos were showed twice to our participants which made them grasp and understand the content of the video as compared to other studies that administered the intervention once. Another possible reason for the improvement in participants' knowledge and awareness of the symptoms and risk factors of cervical cancer could be due to the comprehensive and all-inclusive nature of the content of the video used for the intervention in this study.

Gottvall et al. reported that educational intervention was unable to change participants' intention of getting a pap smear test and the usage of condom (45). Similarly, Kim et al. also reported that educational intervention failed to improve the negative response of high school students toward being screened by pap smear test (16). However, the results of this study contradict the findings of Kim et al. and Gottvall et al. in that, participant's willingness of getting a pap smear test increased in our study after the intervention indicating a positive attitude toward getting a pap smear test. Coronado Interis et al. (47) also reported a similar outcome to our study.

Intriguingly, after the video-based educational study, possible barriers to cervical cancer screening such as “lack of knowledge, poor awareness, lack of understanding about screening procedure, stigma, superstition, fear of embarrassment, anxiety, the pain of pap test and lack of family support" was not changed. This result shows that utilising only educational intervention might not be enough to decrease possible screening barriers. Rosser et al. reported that stigmatisation among women, screening acceptability and cervical cancer risk perceptions failed to decrease after an educational intervention was conducted (53). Similarly, Ebu et al. concluded that the espied barriers toward the screening of cervical cancer remained unchanged in their intervention group (36). The results of the present study are consistent with those mentioned by Ahmed et al. where, there were high perceived barriers among Egyptian women despite intervention implementation (46). Contrary, some studies carried out in advanced nations recorded less post-interventional barriers across the interventional arm. It is logical to believe that well-established schemes to promote cervical cancer screening exist in advanced nations, hence women might not face several obstacles to get a screening test completed.

Nevertheless, screening facilities for cervical cancer might not be adequately developed in some resource-constrained nations like Ghana. Again, details on the availability of screening and in-depth clarification on the procedures for screening, utilising relevant communications (native or local language), could help decrease barriers. Ornelas et al. (54) proposed that culturally tailored educational videos can be used effectively in overcoming resistance to cervical cancer screening.

Our results also revealed the comfortability women had while watching the video in varieties of settings and modalities. For instance, the majority of the participants showed their comfortability in watching the videos as a group and also suggested that the videos must be shown to some women associations or groups within their communities. During a period of several conflicting priorities, consistent and regular messaging utilising a range of modalities can aid ensure that pertinent health information's are received by women. The videos could still be beneficial to women who have previously had a pap smear test since all the participants attested to the fact that they learnt something new, as well as considering that there are sometimes myths regarding cervical cancer screening.

Besides, a major determining factor in the health belief model is the person's confidence level or self-efficacy. Numerous studies have evaluated its influence on screening for cervical cancer (55–57). The results of the current study indicate that higher self-efficacy was observed within participants who were enlightened about cervical cancer screening. This result is in line with the results of an interventional study performed by Del Mistro et al. where the level of self-efficacy within the interventional arm increased significantly in comparison to the control arm (56). Women with improved self-efficacy are more likely to engage in appropriate health-related behaviours because these class of women might have their knowledge status being influenced due to their exposure to some information. Therefore, it is worth remembering that direct mastery experience will significantly improve self-efficacy beliefs (58). Kim et al. concluded that an association exist directly between self-efficacy, health literacy and knowledge levels (59). Taha et al. reported that self-efficacy level increased among diabetic patients after an educational intervention was administered (60). Also, Ndosi et al. concluded that health status and self-efficacy improved significantly after “needs-based patient education” (61). This means that self-efficacy is essential in encouraging people to effectively take action that can ultimately improve their health.

Our findings indicated significant improvements in the willingness to be vaccinated with the HPV vaccine, an outcome that is comparable to other video-based study, accompanied by a group discussion session for preventing Obesity (62). Additionally, the effectiveness of video-based interventions to influence perceptions are evident in the prevention of stroke, HIV-related stigmatisation and risk, and cancer screenings (63–65). Video-based interventions are capable of averting negative attitudes in health-related behaviours. Video-based interventions have the potential to contribute to long-lasting improvements in health-seeking behaviours, nevertheless, in order to achieve these impacts, video-based interventions must be adequately tailored for the specific population of concern. Video-based educational interventions are also effective for training healthcare workers and community health education. Physicians can educate their patients through this means irrespective of language or topic (66–70).

Even though pragmatic measures were put in place to avoid shortfalls and limitations, we still had some limitations. Due to socially perceived value, answers to queries such as willingness-to-be vaccinated and screened may be biased, and might be varied if vaccination and pap smear tests were readily available after the intervention. Again, there was a decrease in our sample size due to the survey being incompletely answered. Nevertheless, we believe that the relevance of our results was not compromised. Additionally, time and budgets constraints did not allow for a longer time of follow-up.

## Conclusion

Utilising an educational intervention based on theory, substantially improved knowledge of cervix carcinoma symptoms, risk factors, Pap smear test and HPV vaccines, which contributed to the majority of the respondents seeking for the Pap smear test and HPV vaccine after the post-intervention. The study results show that health education using videos may be influential in perception changing, self-efficacy improvement and the understanding of cervical carcinoma screening and HPV vaccination. Regardless of the screening barriers observed in the study, the intervention achieved positive belief concerning screening and high level of knowledge. While respondents may be well-educated, and have improved self-efficacy, it was clear that the barriers such as “lack of knowledge, Poor awareness, “lack of understanding about screening procedure, Stigma, Superstition and family support” among others could discourage qualified women from being screened. Initiatives to decrease the barriers may improve the uptake of screening for cervical cancer within the study population. It is important to establish that educational interventions will help women to determine their susceptibility level and implement actions to minimise the likelihood of contracting the disease. The results of this study are vital in steering health educational interventions.

## Data Availability Statement

The raw data supporting the conclusions of this article will be made available by the authors, without undue reservation.

## Ethics Statement

The studies involving human participants were reviewed and approved by Zhengzhou University and Henan Provincial People's Hospital. The patients/participants provided their written informed consent to participate in this study.

## Author Contributions

HA: data curation. KS: funding acquisition and supervision. CA-B: investigation. CE and CA: methodology. ES: software. ED: writing—original draft. ED and GA: writing—review and editing. All authors contributed to the article and approved the submitted version.

## Conflict of Interest

The authors declare that the research was conducted in the absence of any commercial or financial relationships that could be construed as a potential conflict of interest.
